# *In silico* prediction of housekeeping long intergenic non-coding RNAs reveals *HKlincR1* as an essential player in lung cancer cell survival

**DOI:** 10.1038/s41598-019-43758-7

**Published:** 2019-05-14

**Authors:** Danish Memon, Jing Bi, Crispin J. Miller

**Affiliations:** 1 RNA Biology Group, CRUK Manchester Institute, The University of Manchester, Alderley Park, Manchester SK10 4TG UK; 2 Present Address: European Bioinformatics Institute (EMBL-EBI)/Cancer Research UK Cambridge Institute, The University of Cambridge, Cambridge, UK

**Keywords:** Cancer genomics, Transcriptomics, Long non-coding RNAs

## Abstract

Prioritising long intergenic noncoding RNAs (lincRNAs) for functional characterisation is a significant challenge. Here we applied computational approaches to discover lincRNAs expected to play a critical housekeeping (HK) role within the cell. Using the Illumina Human BodyMap RNA sequencing dataset as a starting point, we first identified lincRNAs ubiquitously expressed across a panel of human tissues. This list was then further refined by reference to conservation score, secondary structure and promoter DNA methylation status. Finally, we used tumour expression and copy number data to identify lincRNAs rarely downregulated or deleted in multiple tumour types. The resulting list of candidate essential lincRNAs was then subjected to co-expression analyses using independent data from ENCODE and The Cancer Genome Atlas (TCGA). This identified a substantial subset with a predicted role in DNA replication and cell cycle regulation. One of these, *HKlincR1*, was selected for further characterisation. Depletion of *HKlincR1* affected cell growth in multiple lung cancer cell lines, and led to disruption of genes involved in cell growth and viability. In addition, *HKlincR1* expression was correlated with overall survival in lung adenocarcinoma patients. Our *in silico* studies therefore reveal a set of housekeeping noncoding RNAs of interest both in terms of their role in normal homeostasis, and their relevance in tumour growth and maintenance.

## Introduction

The catalogue of known genes has expanded considerably since the publication of the first draft of the human genome^1^, not only through the detection of additional protein-coding loci^2–4^, but also through the identification of ~22,500 noncoding RNAs (ncRNAs) that do not encode proteins^3^. Of these, approximately two thirds are classified as long intergenic noncoding RNAs (lincRNAs) – a class of transcripts defined solely by their length (>200 nt) and lack of coding potential.

lincRNA function arises directly from the ability to hybridise to specific nucleotide sequences. When binding occurs between molecules, this allows precise targeting of a lincRNA to a given DNA or RNA locus, often through repeat sequences^5,6^. Alternatively, when hybridisation occurs within the same molecule, it supports the establishment of stable structures that lend specificity to interactions with specific proteins^7,8^. Together, these properties allow lincRNAs to perform diverse scaffolding and targeting roles throughout the cell. The primacy of sequence in driving lincRNA function typically results in rapid evolution: a substitution of one base can often be compensated for by a complementary substitution at its binding partner. This differs from protein coding genes, which are under stronger constraints that arise from the need to maintain the complex biochemical properties of a given arrangement of amino acids. Thus, the majority of lincRNAs are less well-conserved than proteins, and undergo only weak positive or neutral selection^6^. This rapid evolution often prohibits the use of phylogenetics for functional annotation, and, when combined with the relative paucity of annotated noncoding genes, means that the majority of lincRNAs have yet to be assigned a function.

The protein coding complement of the genome includes a substantial number of genes that encode basal cellular functions required for cell survival, irrespective of the tissue type or functional role of the cell in question^9^. Here, we refer to these tissue-type and cell-status independent essential genes as ‘housekeeping genes’, following the definition of Eisenberg and Levanon. Housekeepers are important both in terms of their fundamental contribution to the mechanisms that sustain life, but also from the more pragmatic perspective of their utility as experimental controls^9^. Multiple studies have sought to identify housekeeping protein-coding genes^9–19^. These provide significant steps towards more detailed functional characterisation, but work to date has focused primarily on the protein complement of the cell.

With the rapid emergence of lincRNAs as a functionally important and often overlooked class of molecule, an important and unanswered question is the degree to which lincRNAs also serve a critical housekeeping role. Here we use *in silico* methods to generate, and then annotate, an initial list of candidate housekeeping lincRNAs (cHK-lincRNAs). Downstream validation of one such transcript, *HKlincR1* (AC093323.3), confirmed its predicted role in cell survival, thus demonstrating the validity of the approach.

## Results

### Identification of ubiquitously expressed lincRNAs

The Illumina Human BodyMap 2.0 (HBM) RNA-Seq dataset (ArrayExpress: E-MTAB-513) provides a comprehensive catalogue of gene expression encompassing 16 distinct human tissues (adipose, adrenal gland, brain, breast, colon, heart, kidney, liver, lung, lymph node, ovary, prostate, skeletal muscles, testes, thyroid and white blood cells). The comprehensive nature of the dataset makes it particularly valuable when seeking previously uncharacterised noncoding RNAs, particularly in combination with existing annotation databases^20^.

**Figure 1:**
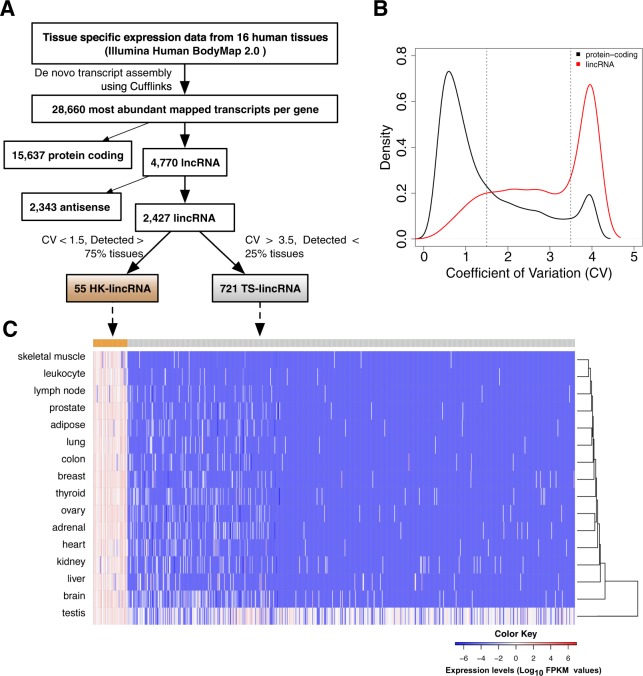
Identification of HK-lincRNAs and TS-lincRNAs from Human BodyMap 2.0 data (**A**) Schematic overview of the computational approach. Cufflinks-assembled transcripts were classified as housekeeping (HK-lincRNA) or tissue specific long intergenic noncoding RNA (TS-lincRNA) according the coefficient of variance (CV) and expression level across 16 different human tissues. (**B**) Distributions of CV for protein coding transcripts and lincRNAs. (**C**) Heatmap of HK-lincRNA and TS-lincRNA expression across 16 tissues. Colours correspond to log_10_ RPKM values. LincRNAs (rows) were ordered by CV of expression across tissues while tissues (columns) were ordered by unsupervised clustering of the expression data.

We first re-annotated the HBM data using Cufflinks to generate *de novo* transcript assemblies and mappings to the reference genome (hg19), with existing gene annotation taken from Ensembl release (v74). A total of 107,651 known transcripts (28,660 unique genes) were detected (FPKM > 0.5; Fig. 1A), including 15,637 protein-coding loci and 4,770 lncRNA genes (2,343 antisense and 2,427 lincRNAs). Since the data were not strand specific, expression measurements for antisense transcripts were less reliable. These were discarded: only lincRNAs > 1 kb from the nearest protein-coding gene were retained for subsequent analysis. In total, 2,427 lincRNAs were considered further.

As expected, lincRNA levels were substantially lower than those at protein coding loci (Supplementary Fig. S1A)^6^. The coefficient of variation (CV) of the normalised transcript levels formed a bimodal distribution, irrespective of gene type (Fig. 1B). While the majority of the protein-coding genes exhibited low CV (over 60% with CV < 1.5), lincRNAs were considerably more variable (less than 25% with CV < 1.5; Fig. 1B).

We reasoned that genes with core essential functions (i.e. housekeepers) would be ubiquitously expressed across human tissues, irrespective of cell type. To test this, we first identified all protein coding genes detected in at least 75% tissues with a CV < 1.5. This set included many genes involved in core essential functions (Gene Ontology (GO) terms: *ncRNA metabolic process*, *protein catabolic process*, *establishment of protein localization of organelle*, *posttranscriptional regulation of gene expression*, *DNA repair*, *oxidative phosphorylation and regulation of cell cycle phase transition*), and overlapped significantly (Fisher’s exact test *p*-value < 0.05) with a set of previously identified housekeeping genes^9^ (Fig. S1B,C). Median CVs for both datasets were similar (Eisenberg: 0.534; HBM: 0.645; data not shown).

Since the behaviour of the protein encoding subset of the data behaved as expected, we then applied the same strategy to lincRNAs. In total, 55 candidate housekeeping lincRNAs (cHK-lincRNAs) were detected in >75% of tissues with a CV < 1.5. Both NEAT1 and NEAT2/MALAT1 were identified by this strategy, in keeping with their critical role in paraspeckle maintenance in the nucleus^21,22^, thus lending further confidence to the strategy. A contrasting set of 721 tissue-specific lincRNAs (TS-lincRNAs; present in <25% of tissues; CV > 3.5) was also defined (Fig. 1A,C; Supplementary Table S1). While cHK-lincRNAs were detected across the tissue panel, many were found at higher levels in ovary (median expression ~4 FPKM) and at lower levels in liver (median expression = ~2 FPKM). The majority of TS-lincRNAs were specific to testis. cHK-lincRNAs also had significantly higher expression levels compared to TS-lincRNAs in ENCODE^23^ cell line data (Fig. S2A), and were more likely to feature CpG islands in their promoters (45% vs. 15%), mirroring previous observations for protein coding housekeepers^12,14^.

### HK-lincRNA sequence and expression patterns are more conserved in mammals than TS-lincRNAs

**Figure 2:**
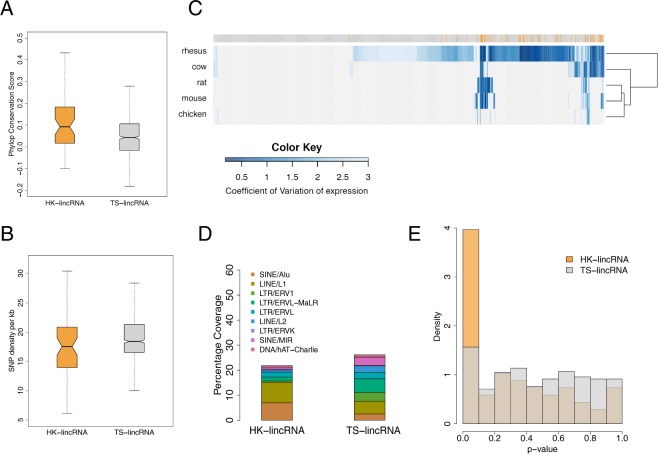
Characterisation of HK-lincRNA and TS-lincRNAs (**A**) Mean PhyloP^24^ conservation scores across exonic nucleotides only, for each lincRNA transcript. (**B**) SNP density (total number of mutations/total length of the exonic region) in HK-lincRNAs and TS-lincRNAs. Mutation data were obtained from dbSNP (v137)^25^. (**C**) Variability of HK-lincRNA and TS-lincRNA homologues. Homologues to human HK- and TS-lincRNAs were identified using nucleotide-BLAST^26^, and expression levels calculated across nine different tissues in five vertebrate species^60^. Heatmap represents an unsupervised clustering of CV values for each lincRNA (columns: orange: HK-lincRNAs; grey: TS-lincRNAs) in each species (rows). (**D**) Annotations of 11 major repeat elements were obtained from Repbase^74^ database to calculate coverage of repeat elements in exonic regions of lincRNA transcripts. (**E**) Minimum free energy (MFE) for each lincRNA was calculated using Randfold^29^ as an indicator of secondary structure stability. Histogram represents distribution of likelihood that each HK-linRNA or TS-lincRNA is more stable than expected by chance. *p*-values were estimated by comparing to a null distribution generated by permuting the sequences (n = 1000), whilst preserving dinucleotide compositions.

We next compared the PhyloP conservation score^24^ of lincRNA exons derived from a 46-way alignment of mammalian genomes for cHK-lincRNAs and TS-lincRNAs. Sequence conservation was significantly higher for cHK-lincRNAs (mean conservation score HK: 0.135 vs TS: 0.065; *p*-value < 0.01; Fig. 2A). Mutation rates derived from dbSNP (v137)^25^ were also considered; cHK-lincRNAs exhibited a marginally lower SNP density (18.5 vs. 19.7 SNPs per gene per kb; *p*-value < 0.05; Fig. 2B).

We then used nucleotide-BLAST^26^ to identify conserved lincRNAs in *de novo* assembled transcriptomes from five other vertebrates (rhesus, cow, rat, mouse, chicken) generated using the same annotation pipeline as before.  Since repeats are an inherent part of lincRNA transcript identity and structure, and thus play a significant role in their function^27,28^, data were not repeat-masked prior to the search. Importantly, since comparisons were performed against transcriptome sequence, repeats in intronic space would not impact upon the scores. Of the 776 human cHK-lincRNAs and TS-lincRNAs, 517 orthologues were found with at least 30% identity. The number of predicted orthologues also decreased progressively with phylogenetic distance, as expected (Fig. 2C). While 54/55 cHK-lincRNAs were expressed in at least one other species, only 64.2% (463/721) of TS-lincRNAs were detected. Further, 81% (44/54) of cHK-lincRNAs exhibited little variation in their expression profiles in all species analysed (median CV = 1; Supplementary Fig. S2B). In contrast, 60% (278/463) of TS-lincRNAs also showed tissue-specific expression profiles in the other vertebrates (median CV = 2). Thus human TS- and cHK-lincRNA are not only conserved, but also display similar expression patterns in other species.

### HK-lincRNAs are enriched for repeat elements with more stable secondary structures than TS-lincRNAs

LincRNA secondary structure is a major determinant of function^27^. Secondary structure stability can be affected by multiple factors including the presence of repeat elements. Since lincRNAs are particularly enriched for repeat elements^5,28^, we compared the repeat distribution of cHK-lincRNAs and TS-lincRNAs. SINE/Alu elements constitute ~7.7% of nucleotides in cHK-lincRNA exons vs. ~2.4% of nucleotides in TS-lincRNA exons (Fig. 2D). This is consistent with previous reports of a positive association between the number of SINE/Alu elements and ubiquitous expression^5^. Next, we used minimum free energy (MFE) to assess the stability of lincRNA secondary structures, computed using Randfold^29^. cHK-lincRNA sequences exhibit significantly lower MFE values (median MFE = −375.18 kcal/mol) than TS-lincRNA (median MFE = −140.93 kcal/mol; *p*-value < 2.9e^−16^). In order to mitigate for the effect of sequence length on MFE values, we compared the MFE value of each lincRNA with a null distribution of MFEs generated by permuting the lincRNA sequence, while maintaining dinucleotide composition. 22% (12/55) of cHK-lincRNAs had significantly lower MFE values than expected (FDR < 0.05; Fig. 2E). No TS-lincRNA was found significant using this approach. Thus cHK-lincRNAs have more stable secondary structures and feature increased representation of repeat elements, in keeping with an active mechanistic role arising from targeted interactions with other molecules in the cell^5,8^.

### HK-lincRNAs are rarely down-regulated or deleted in tumour progression

Recent work has used RNA interference (RNAi) and CRISPR screens to identify a set of “core fitness” protein coding genes, essential for cell viability^30,31^. We hypothesised that these “core fitness” genes would be unlikely to be down-regulated or deleted in human tumours, since loss of expression would negatively impact on growth and/or viability. We first considered mRNA expression levels, interrogating these loci in RNA sequencing data from 13 different tissue types, obtained from the Cancer Genome Atlas (TCGA)^32^. 93% (1497/1580) of these core-fitness genes were expressed in matched normal tissues. Of these, only 19.7% (295/1497) were downregulated relative to the remaining set of “non-core fitness” protein coding genes (58%; 10858/18675) (Wilcoxon rank-sum, *p*-value < 0.001), while 73.7% (1104/1497) were up-regulated in at least one tumour type (absolute fold change (|FC|) > 2; q-value < 0.05; Supplementary Fig. S3A). Thus their expression levels were consistent with the expectation that they would be ubiquitously expressed. We therefore applied the same approach to further refine the set of cHK-lincRNA genes.

**Figure 3:**
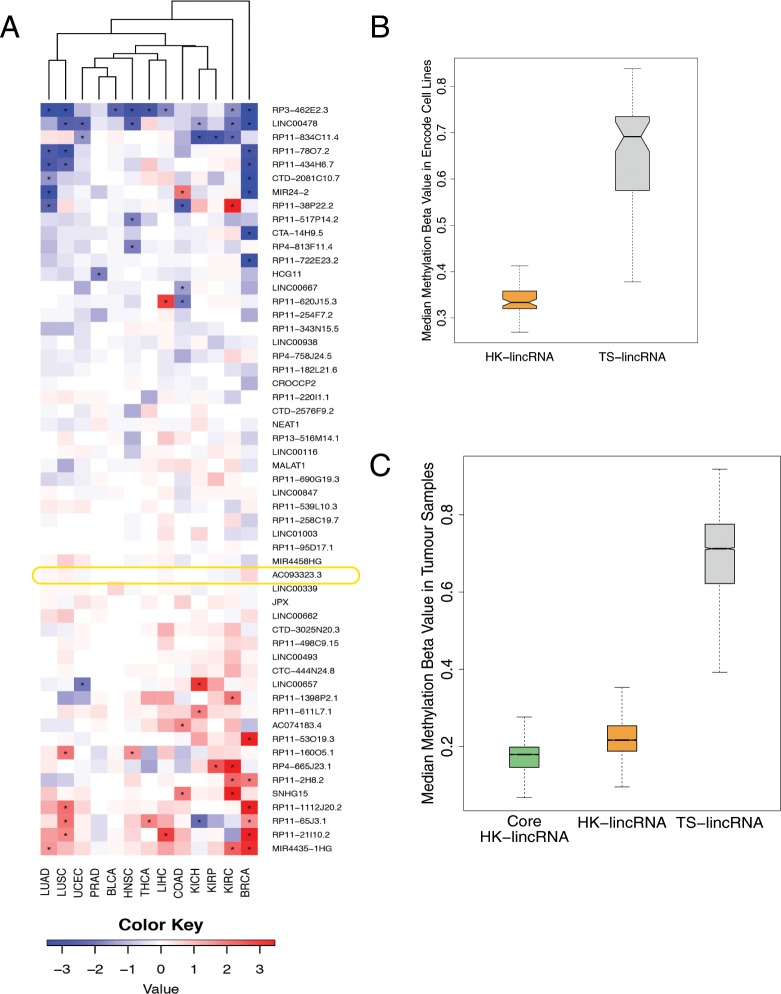
Detection of essential HK-lincRNAs in cancer (**A**) Heatmap showing fold changes of HK-lincRNA expression levels in tumour samples, relative to matched normal samples. Data derived from 13 tumour types from The Cancer Genome Atlas (TCGA)^32^. (**B**) Methylation levels for candidate HK-lincRNAs and TS-lincRNAs. Methylation β values were obtained for 63 Encode cell lines. The median methylation β value per sample was calculated for each group of genes separately. (**C**) Methylation levels for ‘core’ HK-lincRNAs, all candidate HK-lincRNAs and TS-lincRNAs. Methylation β values were obtained for 9,269 TCGA tumour samples. The median methylation β value per sample was calculated for each group of genes separately.

Only 2.3% (212/9142) of lincRNAs were detected across all sample types (FPKM > 0.5). Importantly, these included 72.7% (40/55) of the cHK-lincRNAs identified from the HBM data. Expression levels of 28/55 HK-lincRNAs were significantly altered in one or more tumour type (Cuffdiff |FC| > 2; q-value < 0.05; Fig. 3A). The remaining 27 cHK-lincRNAs were consistently unchanged across all samples. These include JPX, a lincRNA crucial for orchestrating the essential process of X chromosome inactivation during female cell differentiation^33^.

We next tested whether core essential protein-coding genes were less likely to be deleted, using genome-wide copy number data from TCGA representing 31 tumour types. This revealed a small but significant difference in the proportion of core-fitness genes appearing in homozygous deletions (Wilcoxon rank-sum, *p*-value < 0.001). 107/1580 (6.77%) core essential genes and 1,647/17,375 (9.47%) non-core essential genes underwent recurrent homozygous deletions in at least 1% of samples. In contrast, similar proportions of core essential (704/1580; 44.5%) and non- core essential (7651/17375; 44.03%) genes were amplified (homozygous) (Supplementary Fig. S3A).

Having established that previously defined core-fitness protein coding genes were less likely to appear in focal deletions, we then applied the same strategy to cHK-lincRNAs. Beroukhim *et al*.^34^ previously described 76 focal amplifications and 82 focal deletions identified from pooled analysis of copy number alterations across 12 different tumour types. Only 6 cHK-lincRNAs mapped to these focal deletions, providing further evidence in support of their role as *bona fide* housekeeping genes. Together, these stringent analyses identified a list of 34 cHK-lincRNAs that were neither downregulated nor deleted in tumours (Supplementary Table S2). Henceforth we refer to these genes as ‘core’ HK-lincRNAs.

### HK-lincRNAs are less likely to be silenced via DNA methylation

A key mechanism by which cells regulate gene expression is through the methylation of CpG islands in promoter regions. Promoter CpG hyper-methylation is strongly associated with transcriptional repression^35^ and a critical epigenetic mechanism for the transcriptional inactivation of tumour suppressor genes in cancer cells^36^. Reasoning as before, that transcriptional repression of essential genes would negatively impact on cell viability, we compared the methylation levels of cHK-lincRNA and TS-lincRNA in Encode Cell line data (Fig. 3B). cHK-lincRNAs exhibited significantly lower median methylation β values than TS-lincRNAs (p-value < 0.01), indicating that cHK-lincRNAs are rarely hyper-methylated.

Having established that these candidate HK-lincRNAs were consistently subject to patterns of reduced methylation in cancer cell lines, we next investigated their methylation profiles in human tumours. To do this we considered the methylation levels of core-essential protein coding genes in 9,269 TCGA tumour samples profiled using 450 K DNA methylation arrays^32^. Core essential protein coding genes exhibited significantly lower median methylation β values than the non-core subset (p-value < 1e^−16^; Supplementary Fig. S3B), indicating that core essential protein coding genes are rarely hyper-methylated. We then examined candidate HK-lincRNAs. As expected, both candidate HK-lincRNAs and the restricted subset of ‘core’ HK-lincRNAs filtered by expression and copy number, exhibited significantly lower median methylation β values than the non-core subset (p < 10^−16^; Fig. 3C), confirming that core HK-lincRNAs are less likely to undergo transcriptional repression as a consequence of epigenetic regulation of promoter-CpG patterns in tumours.

### Functional prediction of candidate housekeeping lincRNAs

**Figure 4:**
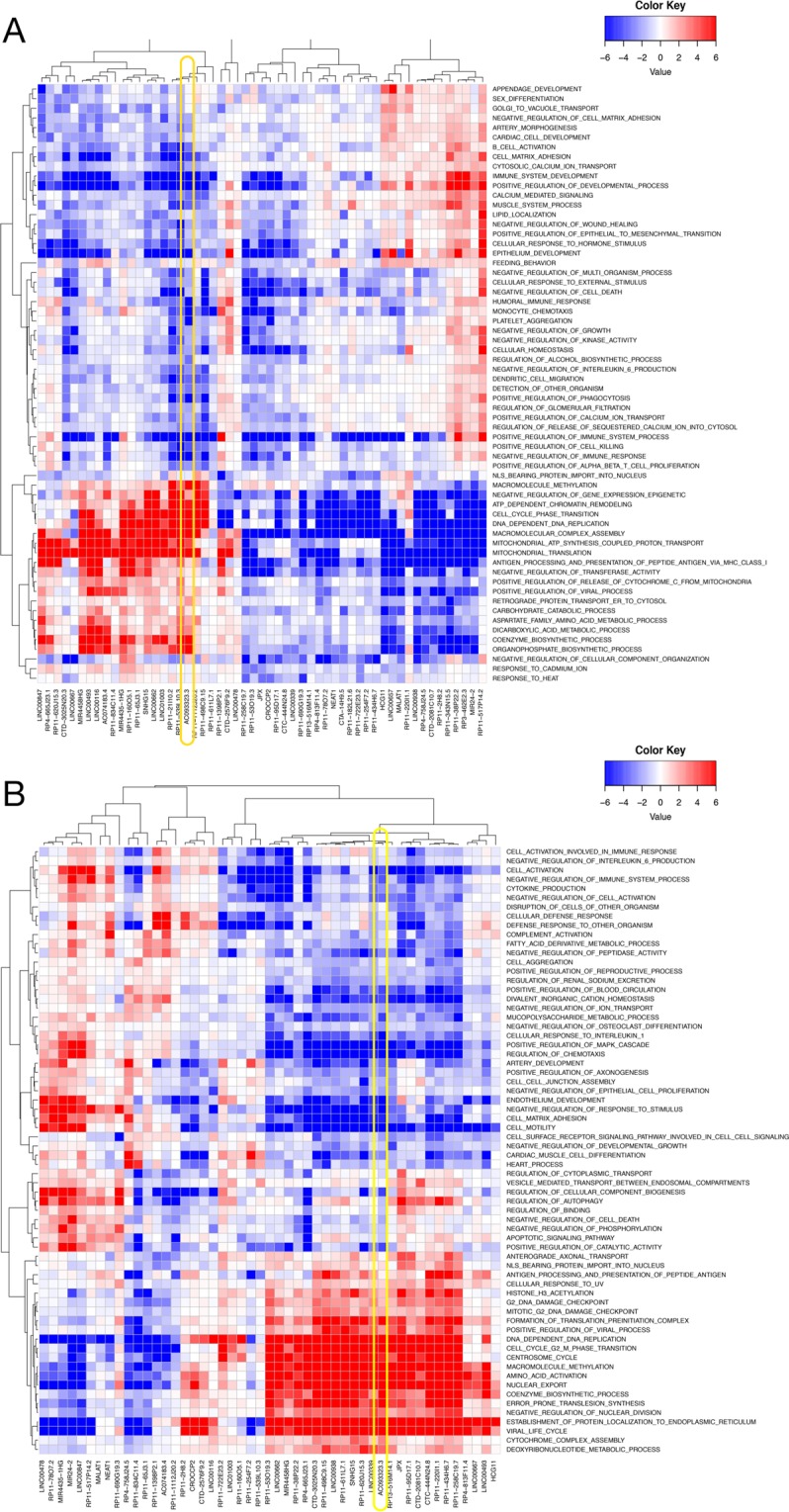
Functional prediction of HK-lincRNAs (**A**) HK-lincRNA function prediction using TCGA lung adenocarcinoma (LUAD)^38^ samples. Rows represent non-redundant Gene Ontology (GO) ‘biological process’ terms. Columns represent each HK-lincRNA. Coloured cells correspond to a significant positive (red) or negative (blue) association between an HK-lincRNA and a biological process. Associations were established based on correlation of expression level of each lincRNA with protein-coding genes. Significance was determined using Gene Set Enrichment Analysis (GSEA)^39^ of protein-coding genes ranked by Pearson correlation to each HK-lincRNA. (**B**) Functional prediction of HK-lincRNA using Encode cell lines. Rows represent non-redundant Gene Ontology (GO) biological processes terms and columns represent HK-lincRNAs. Cells are coloured as red or blue based on significant positive or negative association between HK-lincRNA and a biological process. Significant biological processes (<5% FDR) were identified for each HK- lincRNA using Gene Set Enrichment Analysis (GSEA) of protein-coding genes pre-ranked by the Pearson correlation of gene expression. Only non-redundant biological processes identified using the GOSemSim package are shown.

Many lincRNA genes bear a strong resemblance to canonical protein-coding loci, with similar chromatin marks^37^, PolII mediated transcription, well-defined intron-exon structures, and similar downstream processing including splicing, 5′-capping and 3′ polyadenylation^6^. Together these patterns suggest that lincRNAs are under active regulatory control. We reasoned that lincRNAs with expression profiles similar to sets of functionally related protein-coding genes would be controlled by similar regulatory systems, and thus be involved in similar biological processes. Previous work has successfully used TCGA data as a source of correlative patterns with which to infer noncoding RNA function^20^. We therefore used the TCGA Lung Adenocarcinoma (LUAD) Dataset^38^ comprising 601 samples (542 tumour and 59 normal samples) to calculate gene expression correlations between protein-coding genes and the cHK-lincRNA set. Proteins with significant positive or negative correlations to cHK-lincRNAs were subjected to Gene Set Enrichment Analysis (GSEA)^39^ to identify Gene Ontology Biological Processes showing strong associations to each cHK-lincRNA (Fig. 4A). In an unsupervised analysis, cHK-lincRNAs clustered into two major sub-groups, comprising 30 and 22 HK-lincRNAs, respectively. In the 22 HK-lincRNA cluster, the majority of lincRNAs showed significant positive association with *cell cycle phase transition*, *DNA Dependent DNA replication*, *ATP dependent Chromatin Remodeling*, *Macromolecular Complex Assembly* and other fundamental processes. Finally, applying the same methodology to an Affymetrix Exon array dataset representing 182 ENCODE cell lines^23,40,41^ revealed a striking correspondence in the enriched terms for each cHK-lincRNA (Fig. 4B).

Taken together, these data demonstrate that in these two independent tumour derived cohorts, cHK-lincRNAs are significantly correlated in expression with protein coding genes involved in core essential processes, not only suggesting potential biological roles for these transcripts, but also further corroborating their status as *bona fide* housekeeping genes.

### Loss of *HKlincR1* leads to global changes in transcriptional regulations that are enriched from *in silico* functional prediction

**Figure 5:**
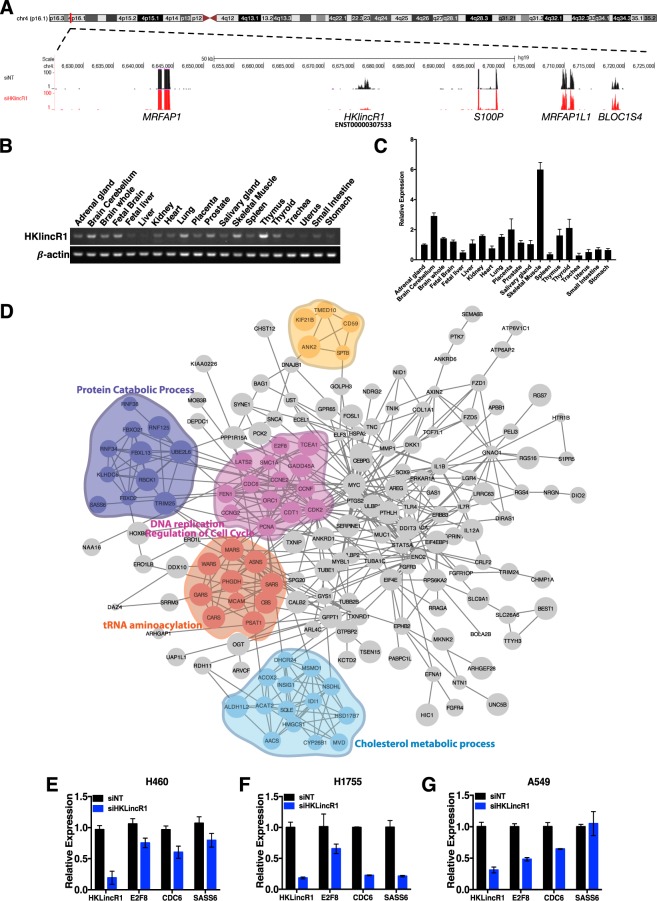
Functional characterisation of *HKlincR1* (AC093323.3) (**A**) Genomic location of *HKlincR1*. Representative image of H460 RNA-seq track encompassing 50 kb up and downstream of *HKlincR1* was plotted using the UCSC genome browser. Non-targeting control track (siNT) was shown in black and si*HKlincR1* track was shown in red. (**B**) RT-PCR and qRT-PCR (**C**) of *HKlincR1* transcript levels across 20 different normal human tissue types. β-actin was used as a positive control. Cropped gel images for *HKlincR1* and β-actin were shown in (A). Full length gels are presented in Supplementary Fig. S4C. (**D**) Protein-protein interaction network of differentially expressed genes following *HKlincR1* knockdown. Significant functional modules were identified using ClusterONE^72^ in Cytoscape^71^ (*p*-value < 0.01; Minimum cluster size >4). Biological processes significantly enriched in each cluster were identified using BiNGO^73^ in Cytoscape (Adjusted *p*-value < 0.05). (**E**–**G**) qRT-PCR analysis of *HKlincR1*-dependent genes relative to β-actin following *HKlincR1* knockdown in H460, H1755 and A549 lung cancer cells. Non-targeting (NT) siRNA was used as a control.

To test the robustness of our computational approaches, we next selected one of the cHK-lincRNAs, *HKlincR1* (AC093323.3), from the 22 HK-lincRNA cluster for further characterisation. *HKlincR1* is located at 4p16.1 (Fig. 5A). *HKlincR1* had relatively high (mean FPKM~3.9) and stable expression (CV~0.59) in the human bodymap dataset. It was neither down-regulated nor deleted across the 13-tissue TCGA tumour cohort, thus belonging to the sub-group of ‘core’ HK-lincRNAs. It also had a very stable secondary structure (MFE = −974.4; FDR < 1%) and was found to have detectable expression in multiple organs of rhesus. *HKlincR1* exhibited strong positive association with the expression of cell-cycle related pathways, and negative association with the stress response, including MAPK signaling (Fig. 4A,B), thus implicating it in key pathways that impact upon proliferation.

We first measured *HKlincR1* levels across 20 different normal human tissues, confirming consistent expression, as expected from the HBM data, and in keeping with its predicted role as a housekeeping lincRNA (Fig. 5B,C). We then used RNA-Seq to profile gene expression changes following a reduction of *HKlincR1* levels in H460 non-small-cell lung cancer cells. siRNA mediated depletion of *HKlincR1* led to a mean 80% reduction in *HKlincR1* across independent triplicate samples and altered expression for a significant number of transcripts (223 up-regulated and 96 down-regulated; Fold change >1.5 and FDR < 0.05; Supplementary Table S3).

Gene set enrichment analysis (GSEA) of REACTOME pathways affected by *HKlincR1* knockdown identified a significant loss of expression in *DNA elongation*, *DNA replication*, and *cell cycle* related pathways (FDR < 0.05; Supplementary Fig. S4A), in keeping with its *in silico* predicted functions from the TCGA and ENCODE datasets (Fig. 4A,B). We next built a protein-protein interaction network capturing known and predicted relationships between the protein-products of the 319 genes differential expressed (DE) following reduced *HKlincR1* expression (STRING database; interactions satisfying a medium confidence cutoff of 400) (Fig. 5D). The network was significantly more connected than expected by chance (*p*-value < 0.01). Functional enrichment analysis of individual modules within the network confirmed functional relationships between adjacent proteins within the network, and revealed similar terms (highlighted in Fig. 5D) to the GSEA REACTOME analysis^39,42^ (Fig. S4A). Finally, we validated a subset of down-regulated genes involved in DNA replication and cell cycle regulation (SASS6, CDC6, and E2F8). In all cases qRT-PCR following *HKlincR1* depletion by siRNA confirmed the RNA-seq results in H460, as well as two other non-small-cell lung cancer cell lines, A549 and H1755 (Fig. 5E–G). Given the considerable overlap between the pathways perturbed following *HKlincR1* depletion and those identified from the TCGA and ENCODE correlation analyses (Fig. 4A,B) thus demonstrates the utility of using *in silico* correlative analyses for functional inference.

### Loss of *HKlincR1* leads to reduced cell viability and is associated with improved patient outcome in lung adenocarcinomas

**Figure 6:**
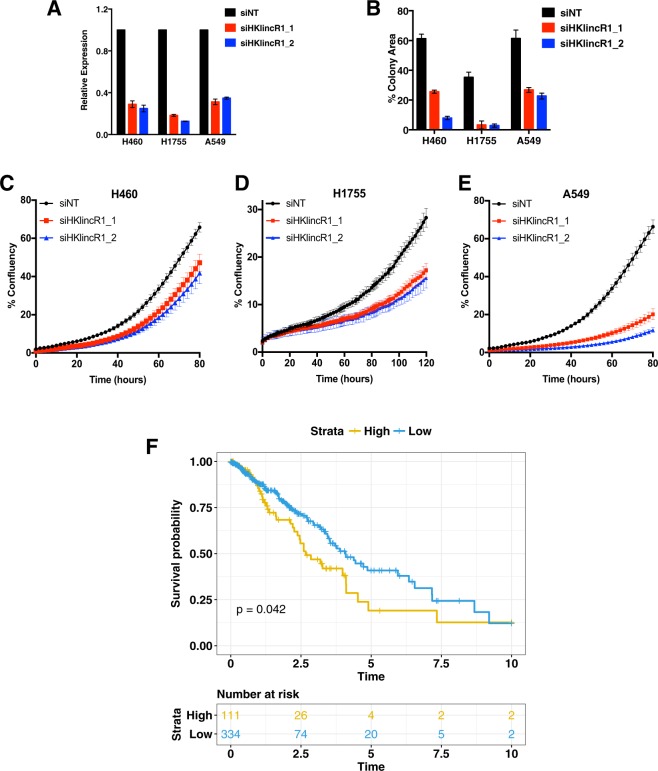
Loss of *HKlincR1* leads to reduced E2F8 levels and altered cell cycle in H460 (**A**) qRT-PCR analysis of *HKlincR1* expression relative to β-actin following *HKlincR1* knockdown in H460, H1755 and A549 using two independent siRNAs. Non-targeting (NT) siRNA was used as a control. (**B**) Cell viability measured by crystal violet assay following *HKlincR1* knockdown using two different siRNAs vs siNT control, in H460, H1755 and A549. (**C**–**E**) Incucyte proliferation assays for H460, H1755 and A549 lung cancer cells. (**F**) Kaplan-Meier plot of overall survival in TCGA LUAD^38^ cohort (N = 464) stratified by *HKlincR1* expression levels, partitioned on the 75^th^ percentile. Blue: upper quartile. Orange: lower 3 quartiles. Kaplan-Meier plot was generated using survival^65^ and survminer packages (based on ggplot2) in R.

We then asked whether the expression of *HKlincR1* is essential for cell survival. Decreased cell viability was observed in H460, A549 and H1755 cells following *HKlincR1* knockdown using two independent siRNAs (Fig. 6B). Furthermore, all three cell lines exhibited impaired proliferation following loss of *HKlincR1* expression (Fig. 6C–E). Given the association between *HKlincR1* and cell survival, we asked whether altered *HKlincR1* levels were associated with overall survival in the TCGA LUAD data. *HKlincR1* levels were significantly correlated with poor outcome (Fig. 6F).

## Discussion

Given their emerging importance in regulating multiple functions across the cell, it is not surprising that deregulation of lincRNA expression is linked to many diseases including cancer^20,43–45^, however, despite rapid progress in the field, a fundamental question that remains unclear is how many are required for growth and viability.

Here we present a systematic study of these housekeeping lincRNAs. We identify lincRNA genes conserved both in sequence and tissue expression patterns across 6 vertebrate species (human, rhesus, cow, rat, mouse, and chicken). Subsequent analyses were then used to refine this set by reference to human tumour data, since loss of *bona fide* housekeepers would be expected to negatively impact on growth and/or viability, and thus be selected against in tumours. In parallel, we were able to validate each analytical step by reference to protein coding genes: applying the same filtering method to the protein coding transcriptome successfully enriched for known housekeepers, as expected. These analyses produced a set of candidate housekeeping lincRNAs (cHK-lincRNAs) ubiquitously expressed and rarely deleted, downregulated, or subject to epigenetic repression across a multitude of tumour tissue types.

We were then able to use tumour expression data from two independent datasets to infer biological roles for each candidate housekeeper, by exploiting the fact that co-expression is often indicative of common patterns of regulation and control, and thus, of common function^46–49^. This strategy has recently been used successfully to characterize lincRNAs with an inferred role in cancer^20^. As expected, HK-lincRNAs were significantly associated with protein coding genes involving core essential tasks including DNA replication, metabolism and cell cycle control.

These correlation analyses are also valuable because the rapid evolution of ncRNA sequence places particular demands on genome annotators^50^, since approaches that attempt to predict function by establishing orthology or parology between lncRNA genes are generally thwarted by the rate at which sequences diverge. These challenges are further exacerbated by the fact that the vast majority of lncRNAs have yet to be assigned a function. Thus, even if a relationship can be established *in silico*, it is unlikely that either gene will have a known function or mechanism associated with it. This paucity of annotation is therefore a major barrier to progress, since without knowing broad mechanism, it is hard to predict which experimental techniques are most likely to yield dividends, and which pathways and phenotypes are most likely to be effected by loss- and gain of function assays.

In order to ascertain the validity of our *in silico* annotations we selected one candidate, *HKlincR1*, for downstream validation. As well as passing the series of filters described above, *HKlincR1* was not only detected across normal human tissue array but its expression is essential for the cell survival and high expression levels were also predictive of poor outcome in TCGA lung cancer samples. Depletion of *HKlincR1* led to altered expression of several cell growth and division related genes including CDC6, SASS6 and E2F8, however, the mechanism of regulation of these genes is unclear. Bioinformatics analysis using *iRegulon*^51^ to search for potential regulators of *HKlincR1* dependent genes indicates E2F transcription factor 8 (E2F8) as a significant candidate (*p*-value < 0.01). In addition, E2F8 target genes which include CDC6, SASS6 and E2F8 itself, were significantly enriched among differentially expressed genes following *HKlincR1* knockdown (Gene Set Enrichment NES Score = −1.41; *p*-value < 0.005) in an independent E2F8 ChIP-Seq dataset from lung cancer cell lines^52^ (Supplementary Fig. S4B). These preliminary analyses may thus indicate a direct or indirect relationship between the E2F8 transcription factor and downstream targets of *HKlincR1* in H460. In addition, the significant association between *HKlincR1* and overall survival confirms the association between *HKlincR1* expression and human disease. It therefore serves to highlight the importance of considering lincRNAs when seeking candidate tumour suppressors and oncogenes with a direct impact upon disease progression.

Recently, it has been shown that lincRNAs can express short peptides^52^. While many of these are unlikely to be functional, some do perform a mechanistic role (e.g.)^53^, thus further elevating the importance of lincRNA genes as critical effectors within the genome. Interestingly, despite the fact that *HKlincR1* has low coding potential (Table S2) it has been identified as a ribosome associated lincRNA^54^. In addition, there is also some evidence in the most recent version of ENSEMBL (v95) that the *HKlincR1* locus includes a transcript with the potential to encode a protein product^55^, in keeping with the possibility that it is also a microprotein-expressing lincRNA. Similarly, the candidate housekeeping lincRNA *LINC00116* (Table S2) has recently been shown to express a 56 amino-acid mitochondrion-associated microprotein, Mtln^56^, implicated in respiratory chain assembly, and thus of fundamental importance. Together these data demonstrate the value of *in silico* analyses, not only for the identification and annotation of noncoding RNAs, but also as means by which to infer biological function. This is particularly useful given the relatively small number of lincRNAs that have so far been characterised.

## Materials and Methods

### Cell culture and siRNA transfections

Non-small-cell lung cancer cell lines, including H460, H1755 and A549 were purchased from ATCC. The cells were cultured in RPMI-1640 supplemented with 10% fetal bovine serum, passaged every three days, and tested routinely for mycoplasma contamination. For knockdown experiments, 50 nM siRNAs were transfected using the Lipofectamine RNAiMAX transfection reagent (ThermoFisher, 13778075) for 48 hours before sample collection. siRNAs were purchased from Dharmacon with ON-TARGETplus modification. siRNA sequences used were as follows: si*HKlincR1*_1, GCGGAUGACUUCAGCAUUA; si*HKlincR1*_2, GAAGUAUACUCGUGUGCUU. ON-TARGETplus Non-targeting (NT) siRNA #1 (Dharmacon, D-001810-01-05) was used as a negative control. As the two siRNAs achieved similar knockdown efficiency in H460 cells, only one of the siRNA (si*HKlincR1*_2) was used in the RNA-seq experiments.

### Cell viability and proliferation assay

Cell viability was measured by crystal violet assay 48 hours after the siRNA transfection. In brief, the plate was washed with PBS, fixed with ice-cold methanol, stained with 2% crystal violet solution in 25% methanol and photographed. The areas covered by stained cells were quantified by Image J. The proliferation assay was performed using the IncuCyte® S3 live-cell analysis platform. 48 hours after the siRNA transfection, the cells were collected and 800 cells (H460 and A549) or 2000 cells (H1755) were seeded in 96-well plates. Cell confluence was measured every two hours and quantified by the IncuCyte® imaging system (Essen Bioscience).

### Quantitative RT-PCR analysis

Total RNA from cell lines were extracted using the RNeasy mini kit (QIAGEN, 74104) and reverse transcribed using the M-MLV reverse transcriptase (Promega, M1701). Human total RNA from 20 different tissue sites was purchased from Clonetech, 636643. Expressions were quantified by semiquantitative and quantitative PCR (Fast start SYBR green, Roche, 04673484001) and shown as normalised expression relative to beta-actin. Error bars represent the standard deviations of the average expression based on three biological replicates. The primers used were as follows: HKlincR1 (FW 5′-GCCTGCGTTTTCTCCACATT-3′; RE 5′-GCAGCAGCGTACGTACTGTA-3′), SASS6 (FW 5′ –CCCTCATGATTTTCAGGTGTTGA-3′; RE 5′-ACTAAAACCTGCTCATAACCTCA-3′), CDC6 (FW 5′-AACCTATGCAACACTCCCCA-3′; RE 5′-TTGTTTTGGTGAACTTTGGCT-3′), E2F8 (FW 5′-TTTGGAACCACTGTCCTCGA-3′; RE 5′-ACAGATGCCACCACTGAGAA-3′).

### Dataset description

LincRNA expression measurements were obtained from the publically available Illumina Human Body Map (HBM) RNA-seq set generated from the Human BodyMap 2.0 Project. This dataset comprises of RNA-seq data obtained from 16 human tissues: adipose, adrenal gland, brain, breast, colon, heart, kidney, liver, lung, lymph node, ovary, prostate, skeletal muscles, testes, thyroid and white blood cells with an average of 160 million reads sequenced from each tissue. High read depth is critical for non-coding RNAs, which tend to be more lowly expressed as compared to their coding counterparts.

### Processing of BAM files

The BAM files for the HBM Dataset, comprising of 50mer paired reads aligned to the human genome (hg19) using TopHat (v2)^57^, were downloaded from the UCSC Browser^58^. Transcript models were derived for each sample independently using Cufflinks^59^ (v2.2.0; with default parameters). Resultant models were then merged using Cuffmerge to provide a global model and to classify transcripts as novel, or known, when they mapped to ENSEMBL^3^ (v74). For each gene, we identified the most abundant (highest mean expression) ‘known’ transcript and thus ended up with only 28,660 transcripts.

### LincRNA Conservation, Mutation and Secondary Structure

The nucleotide-level repeat masked conservation scores^24^ for human (hg19) were obtained from the ‘phyloP46wayPrimates’ track in the UCSC database (http://genome.ucsc.edu/index.html). Mutation data was obtained from dbSNP^25^ database (http://www.ncbi.nlm.nih.gov/snp). Both the conservation and mutation data was intersected with lincRNA exon annotations in Ensembl (v74) using bedtools (http://code.google.com/p/bedtools/), which allowed us to make inferences about differences between HK-lincRNA and TS-lincRNA in terms of conservation rates and mutation density. Calculation of minimum free energy from lincRNA transcript sequences and estimation of *p*-value from MFE distribution was performed using Randfold^29^. For each sequence the MFE value was compared against a null distribution generated by repeated shuffling of the sequence without changing the dinucleotide composition. For lincRNA conservation in other species, raw expression data from nine organs (brain, colon, heart, kidney, liver, lung, muscle, spleen, testes) of five species (rhesus, mouse, rat, cow, chicken)^60^ were downloaded and subjected to *de novo* transcript assembly and quantification using Cufflinks and the corresponding Ensembl (v74) genome annotation as guide.

### Gene set enrichment analysis (GSEA) for lincRNA function prediction

HK-lincRNA function prediction was performed using publically available TCGA LUAD dataset comprising of expression measurements from 542 tumour samples and 59 normal lung samples. For each HK-lincRNA, we calculated correlation to all protein coding genes. Protein coding genes were rank-ordered according to these correlation coefficients and GSEA analysis was then performed using the Pre-Ranked tool in javaGSEA3.1 to seek enriched GO biological processes (c5 category from MSigDB). The gene ontology terms list was subject to a number of filtration steps. We selected terms that were found to be significant in at least 10% of HK-lincRNAs. For ease of analysis, the significant gene ontology terms list was further refined to retain only non-redundant terms. GO term similarity was calculated using the GOSemSim^61^ package in R. The GO semantic similarity matrix was used to construct a tree, which was cut at level (h = 0.85) to define GO clusters. From each GO cluster, we randomly selected one from each of the many similar GO terms. GSEA based function prediction was also performed using a publically available Exon Array dataset (GSE19090) comprised of expression measurements from 182 ENCODE cell lines (tier 1, tier 2 and tier 3 cell types). The Affymetrix GeneChip Human Exon 1.0 ST Array features reliable probesets targeting 50 out of 55 HK-lincRNAs. Reliable probesets were then mapped to the ENSEMBL human genome annotation (v74) using the annmap Bioconductor package^62^ and expression for each gene was calculated based on the median expression levels of all probesets mapped to the gene. Non-redundant gene ontology terms were identified using the same approach as that for the TCGA LUAD Dataset.

### Analysis of TCGA data

Raw RNA-seq data with matched normal and tumour samples were obtained from TCGA for 13 different tumour types (BLCA – Bladder Urothelial Carcinoma, BRCA – Breast invasive carcinoma, COAD – Colon Adenocarcinoma, HNSC – Head and Neck squamous cell carcinoma, KICH – Kidney Chromophobe, KIRC – Kidney renal clear cell carcinoma, KIRP – Kidney renal papillary cell carcinoma, LIHC – Liver Hepatocellular Carcinoma, LUAD – Lung adenocarcinoma, LUSC – Lung squamous cell carcinoma, PRAD - Prostate Adenocarcinoma, THCA – Thyroid carcinoma, UCEC - Uterine Corpus Endometrial Carcinoma). These data was used to estimate gene and transcript abundance based on human genome annotations in Ensembl (v74) following the same steps as the HBM Dataset. Differential expression analysis was performed using Cuffdiff^57,59,63^. Genes/transcripts were considered as differentially expressed if they showed at least 2-fold differences in expression between the normal and tumour samples, with a *q*-value < 0.05. Copy number analysis was performed using 10,654 samples from 31 tumour types currently available in TCGA. Processed copy number data was obtained from cBioPortal database^64^. For each sample, these data comprise of gene-level assignment of copy number values into one of 5 categories: homozygous deletion, heterozygous deletion, diploid, heterozygous amplification and homozygous amplification. The proportion of copy number aberrations was compared between core essential and other (non-core essential) protein–coding genes. For survival analysis, clinical annotations were obtained for 446 lung adenocarcinoma samples from TCGA with overall survival data. Kaplan-Meier analysis was performed and plotted using the survival^65^ and survminer packages in R.

### RNA sequencing

Total RNA from three biological replicates of siNT and siHKlincR1 using H460 cells were sent for PolyA sequencing. Indexed PolyA libraries were prepared using 200 ng of total RNA and 14 cycles of amplification with the Agilent SureSelect Strand Specific RNA Library Prep Kit for Illumina Sequencing (Agilent, Cat No: G9691A). Libraries were quantified by qPCR using a Kapa Library Quantification Kit for Illumina sequencing platforms (Kapa Biosystems Inc., Cat No: KK4835). Paired-end 75 bp sequencing was carried out by clustering 1.9 pM of the pooled libraries on a NextSeq. 500 sequencer (Illumina Inc.).

### Analysis of differential expression in knockdown samples

All statistical analysis including t-tests and Wilcoxon’s tests, were performed in R. Gene-level counts of HK-lincRNA-knockdown and scrambled siRNA treated samples generated as biological triplicates were obtained using the gene models in Ensembl (v74) (4) and Rsubread package^66^. edgeR was used to identify differentially expressed transcripts between *HKlincR1* knockdown samples relative to the NT siRNA control^67,68^. An FDR threshold of 0.05 was used for differential expression analysis, with a relatively permissive fold change threshold of 1.5 chosen in order to better support subsequent Gene Set Enrichment Analysis (GSEA). GSEA^39^ was performed on the control vs treatment samples using javaGSEA3.1 and GO REACTOME gene sets (c2.cp.reactome.v6.1.symbols.gmt: c2 category from MSigDB^69^). Gene sets with absolute NES score > 1.7 and FDR < 10% were considered significant. Interactions between differentially expressed protein-coding genes were obtained from the STRING database^70^. Network analysis of protein-protein interactions was performed using Cytoscape^71^. The network was searched for significant functional modules with high intra-module interactions using ClusterONE^72^ in Cytoscape. Modules with at least 5 proteins and *p*-value < 0.01 were considered significant. Biological processes significantly enriched in each module were then identified using BiNGO^73^ in Cytoscape (Adjusted P-value < 0.05). Significant upstream regulators (*p*-value < 0.01) of differentially expressed genes were predicted using the iRegulon tool^51^, which investigates known transcription factor (TF) motifs in upstream regions of genes and genome binding regions of TFs from previously published ChIP-Seq datasets. ENSEMBL v74 and v95 gene symbols in Table S2, were resolved against ENSEMBL stable gene and transcript IDs using BioMart.

### Methylation data analysis

#### ENCODE data

Processed ENCODE/HAIB DNA Methylation data were obtained for 63 cell lines (GSE40699) from UCSC portal. The dataset comprised of Methylation Beta values for nearly 450 K probes generated using the Illumina 450 K array. For each sample, gene level methylation was estimated by taking the mean of the probe-level signal for all probes falling within the promoter region and beginning of gene body (−2000 bp to 200 bp) based on gene models in Ensembl (v74). Probe annotations were obtained using FDb.InfiniumMethylation.hg19 R package. The gene-level Methylation Beta values were used to make comparisons between different categories of genes.

#### TCGA data

Processed probe-level DNA methylation Illumina 450 K array data for 9,269 tumour samples belonging to 33 tumour types was obtained using GDC Data Portal Legacy Archive in TCGA on 20/09/2018. The dataset analysis pipeline was as described above for the Encode Methylation dataset.

## Supplementary information

Supplementary table legends

Table S1

Table S2

Table S2

Supplementary Materials

## Data Availability

H460 cell line RNA sequencing data is available from the Gene Expression Omnibus (GEO Accession: GSE115659). Permission was obtained to use the raw data from TCGA under the project #8211: “Identification of noncoding tumour suppressors and oncogenes”. The results published here are part based upon data generated by TCGA managed by the NCI and NHGRI. Information about TCGA can be found at http://cancergenome.nih.gov.
