# Publisher Correction: *In silico* prediction of housekeeping long intergenic non-coding RNAs reveals *HKlincR1* as an essential player in lung cancer cell survival

**DOI:** 10.1038/s41598-019-55753-z

**Published:** 2019-12-24

**Authors:** Danish Memon, Jing Bi, Crispin J. Miller

**Affiliations:** 1 RNA Biology Group, CRUK Manchester Institute, The University of Manchester, Alderley Park, Manchester, SK10 4TG UK; 2 Present Address: European Bioinformatics Institute (EMBL-EBI)/Cancer Research UK Cambridge Institute, The University of Cambridge, Cambridge, UK

Correction to: *Scientific Reports* 10.1038/s41598-019-43758-7, published online 14 May 2019

A supplementary file containing figures S1-S4 was omitted for the original version of this Article.

Additionally, Figure 3B was incorrectly labelled as BB.

These errors have now been corrected in the HTML and PDF versions of this Article.

