# Supplementary material for: In silico prediction of housekeeping long intergenic non-coding RNAs reveals HKlincR1 as an essential player in lung cancer cell survival
Source: Sci Rep. 2019 May 14;9:7372. doi: 10.1038/s41598-019-43758-7 (PMC6517443; doi:10.1038/s41598-019-43758-7)
Supplement: Supplementary file 1 — Supplementary table legends [file 41598_2019_43758_MOESM1_ESM.pdf]

***In silico* prediction of housekeeping long intergenic non-coding RNAs reveals *HKlincR1* as an essential player in lung cancer cell survival**

Danish Memon [§,$], Jing Bi [§], Crispin J Miller *

RNA Biology Group, CRUK Manchester Institute, The University of Manchester, Alderley Park, Manchester SK10 4TG UK

*corresponding author: crispin.miller@cruk.manchester.ac.uk

[$]current address: European Bioinformatics Institute (EMBL-EBI) / Cancer Research UK Cambridge Institute, The University of Cambridge, UK.

[§]equal contribution

Supplementary Tables

Supplementary Table S1
List of HK-lincRNA and TS-lincRNA identified from the HBM RNA-Seq dataset.

Supplementary Table S2
List of ‘core essential’ HK-lincRNAs.

Supplementary Table S3
Differentially expressed protein-coding genes on knockdown of HKlincR1.
