# Supplementary material for: In silico prediction of housekeeping long intergenic non-coding RNAs reveals HKlincR1 as an essential player in lung cancer cell survival
Source: Sci Rep. 2019 May 14;9:7372. doi: 10.1038/s41598-019-43758-7 (PMC6517443; doi:10.1038/s41598-019-43758-7)
Supplement: Supplementary file 5 — Supplementary Materials [file 41598_2019_43758_MOESM5_ESM.pdf]

Supplementary Materials

In silico prediction of housekeeping long intergenic non-coding RNAs reveals *HKlincR1* as an essential player for lung cancer cell survival

Danish Memon [§,$], Jing Bi [§], Crispin J Miller *

RNA Biology Group, CRUK Manchester Institute, The University of Manchester, Alderley Park, Manchester SK10 4TG UK

* corresponding author: crispin.miller@cruk.manchester.ac.uk

[$] current address: European Bioinformatics Institute (EMBL-EBI) / Cancer Research UK Cambridge Institute, The University of Cambridge, UK.

[§] equal contribution

Supplementary Figures

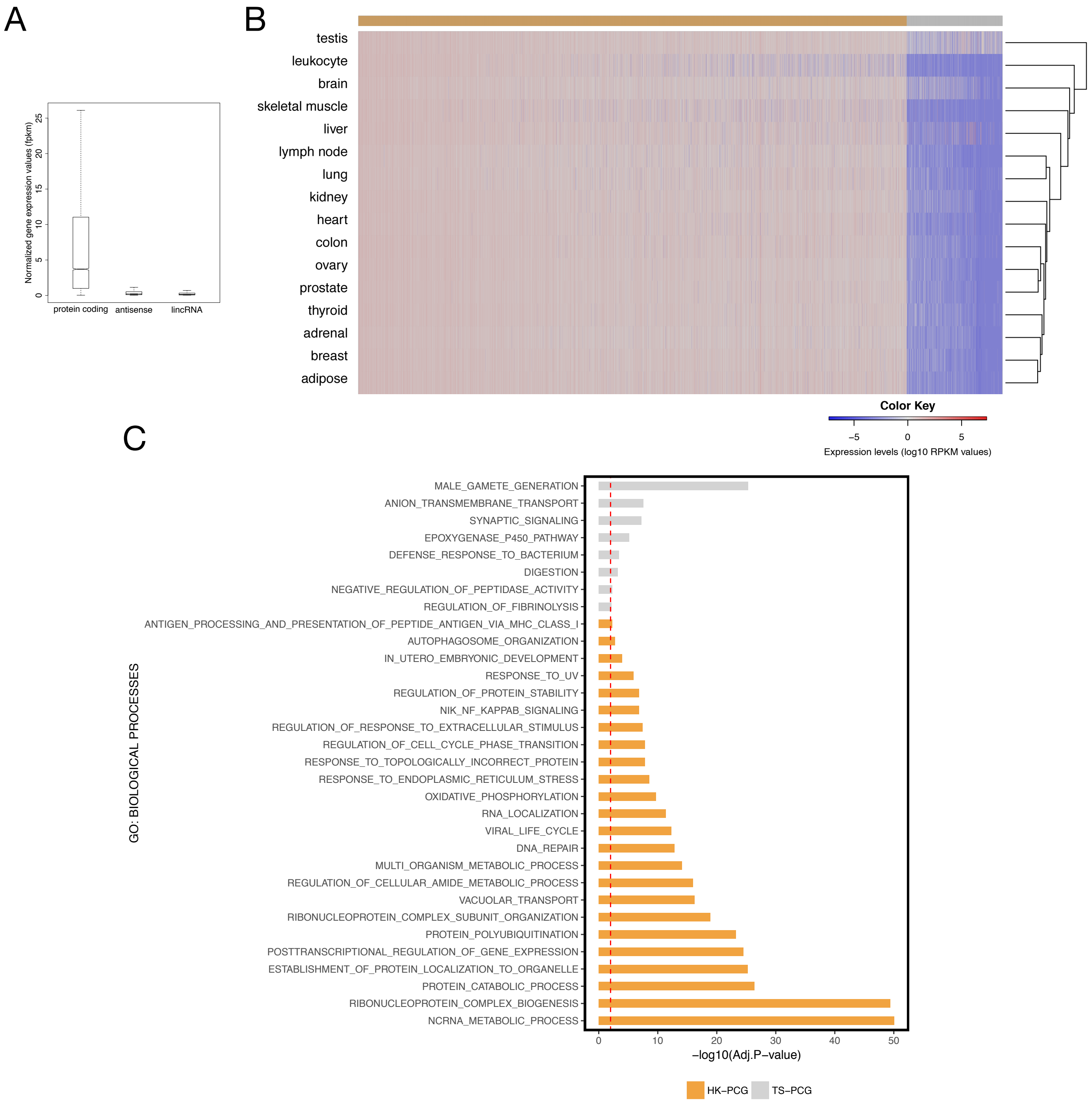

Figure S1: (A) Comparison of abundance of protein-coding transcripts, antisense transcripts and lincRNAs in the Human Body Map dataset. **(**B) Heatmap of HK-mRNA and TS-mRNA expression across 16 tissues. Colours correspond to $\log_{10}$(RPKM). Protein-coding genes (rows) were ordered by CV of expression across tissues. Tissues (columns) were ordered by unsupervised clustering of the expression data. (C) Over-representation analysis of housekeeping protein-coding genes. Protein-coding genes were classified into two groups: housekeeping i.e. (HK-mRNAs with CV < 1.5) and tissue-specific (TS-mRNAs with CV > 3). Each group was then subjected to Gene Ontology Enrichment Analysis (GOA) of Biological Processes from MSigDB (1) using the clusterProfiler (2) package in R. Non-redundant biological processes identified using GOSemSim (3) package are shown.

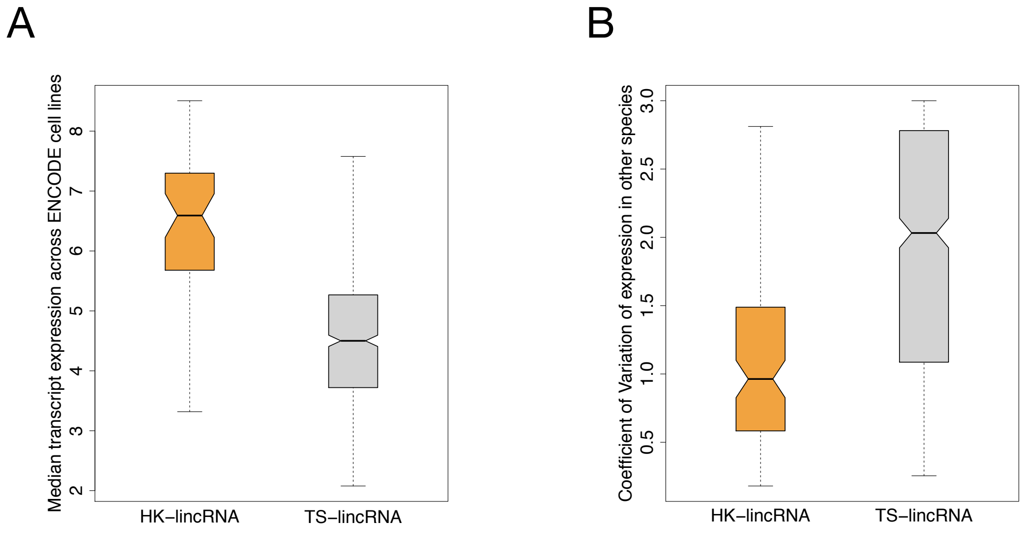

Figure S2: (A) Comparison of HK-lincRNA and TS-lincRNA abundance in Encode cell lines. Median expression of each transcript was calculated across cell lines. (B) Comparison between the CV of expression for HK-lincRNAs and TS-lincRNAs. Coefficient of variation of lincRNA expression was calculated separately for each species from expression data of nine organs (previously published by (4)).

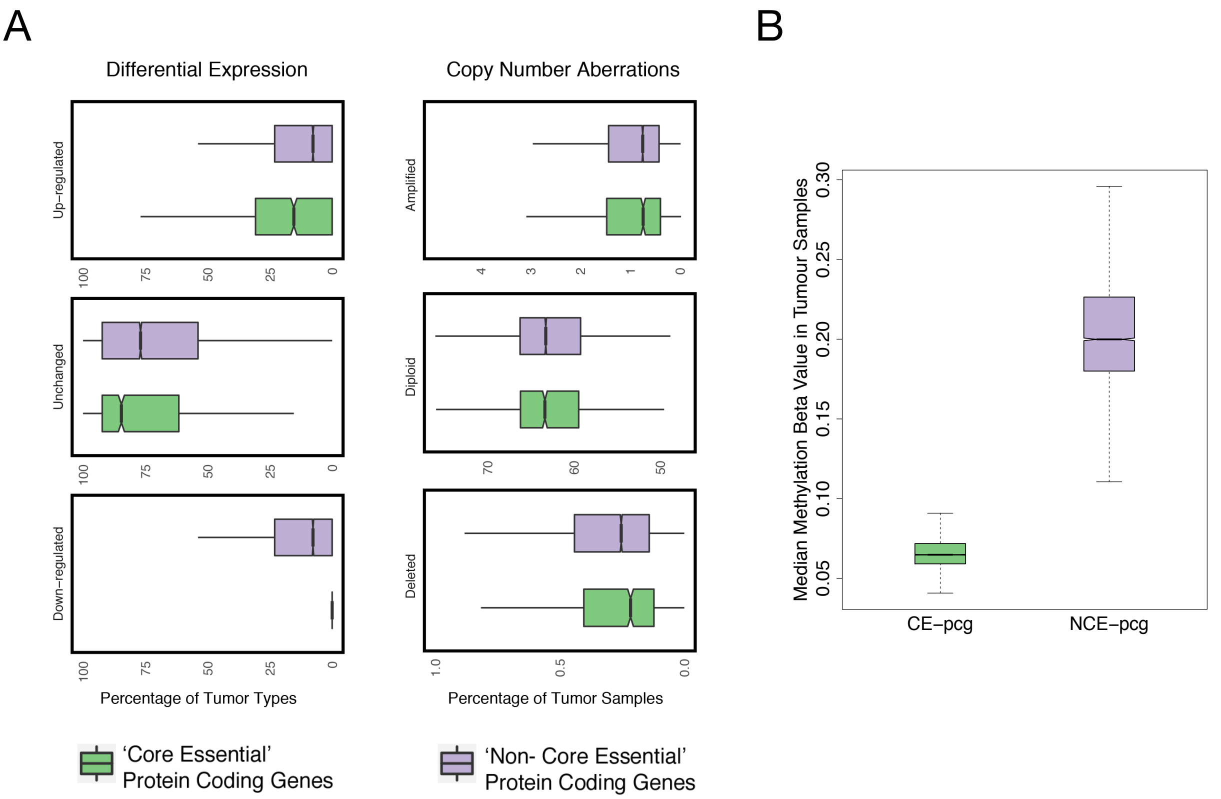

Figure S3: (A) Comparison between expression changes and copy number profiles of ‘core essential’ and the remaining (‘other’) protein-coding genes using multiple tumour types. 1580 ‘core fitness’ protein-coding genes reported by (5) were classified as ‘core essential’ genes. These were compared to the remaining protein-coding genes. Differentially expressed genes (absolute fold change (|FC|) > 2 and q-value < 0.05) were identified for 13 tumour types. Copy number analysis was performed for 31 tumour types in TCGA. Processed copy number data was obtained from the cBioPortal database (6). (B) Methylation levels for ‘core’ protein-coding genes and other protein-coding genes. Methylation β values were obtained for 9,269 TCGA tumour samples. The median beta methylation value per sample was calculated for each group of genes separately.

A

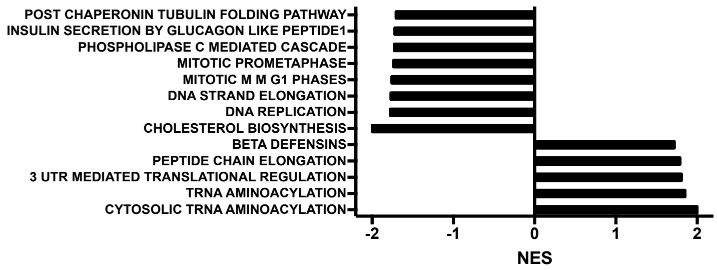

B

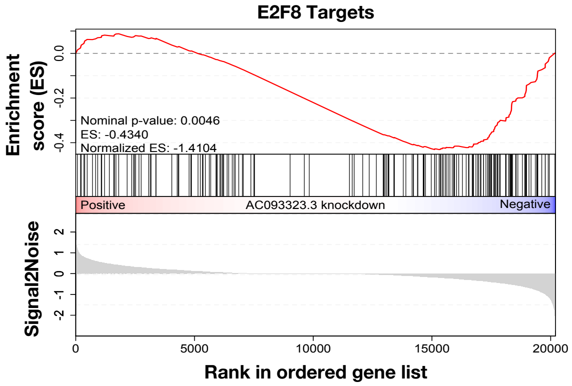

Figure S4: (A) Enriched REACTOME pathways following knockdown of *HKlincR1* in H460 cells. Significant pathways were identified by GSEA (7) on protein-coding genes pre-ranked by fold changes in expression between knockdown and non-target cells (FDR < 10%, abs. NES Score > 1.7). (B) GSEA analysis of E2F8 target gene expression changes following knockdown of *HKlincR1*. E2F8 targets were obtained from previously published ChIP-Seq Study in Lung cancer cell lines (8).

References

1. Liberzon,A., Subramanian,A., Pinchback,R., Thorvaldsdóttir,H., Tamayo,P. and Mesirov,J.P. (2011) Molecular signatures database (MSigDB) 3.0. *Bioinformatics*, **27**, 1739–1740.
2. Yu,G., Wang,L.-G., Han,Y. and He,Q.-Y. (2012) clusterProfiler: an R Package for Comparing Biological Themes Among Gene Clusters. *Omi. A J. Integr. Biol.*, **16**, 284–287.
3. Yu,G., Li,F., Qin,Y., Bo,X., Wu,Y. and Wang,S. (2010) GOSemSim: An R package for measuring semantic similarity among GO terms and gene products. *Bioinformatics*, **26**, 976–978.
4. Merkin,J., Russell,C., Chen,P. and Burge,C.B. (2012) Evolutionary Dynamics of Gene and Isoform Regulation in Mammalian Tissues. *Science (80-.).*, **338**, 1593–1599.
5. Hart,T., Chandrashekhar,M., Aregger,M., Steinhart,Z., Brown,K.R., MacLeod,G., Mis,M., Zimmermann,M., Fradet-Turcotte,A., Sun,S., *et al.* (2015) High-Resolution CRISPR Screens Reveal Fitness Genes and Genotype-Specific Cancer Liabilities. *Cell*, **163**, 1515–1526.
6. Gao,J., Aksoy,B.A., Dogrusoz,U., Dresdner,G., Gross,B., Sumer,S.O., Sun,Y., Jacobsen,A., Sinha,R., Larsson,E., *et al.* (2013) Integrative analysis of complex cancer genomics and clinical profiles using the cBioPortal. *Sci. Signal.*, **6**.
7. Subramanian,A., Tamayo,P., Mootha,V.K., Mukherjee,S., Ebert,B.L., Gillette,M.A., Paulovich,A., Pomeroy,S.L., Golub,T.R., Lander,E.S., *et al.* (2005) Gene set enrichment analysis: A knowledge-based approach for interpreting genome-wide expression profiles. *Proc. Natl. Acad. Sci.*, **102**, 15545–15550.
8. Park,S.-A., Platt,J., Lee,J.W., Lopez-Giraldez,F., Herbst,R.S. and Koo,J.S. (2015) E2F8 as a Novel Therapeutic Target for Lung Cancer. *J. Natl. Cancer Inst.*, **107**, 1–16.
